# Developing bullying prevention guidelines for nurse interns’ and its effects on their assertiveness

**DOI:** 10.1186/s12912-024-02128-z

**Published:** 2024-07-16

**Authors:** Lobna S. Abou El yazied, Rabab M. Hassan, Fawzia M. Badran

**Affiliations:** Nursing Administration Department, Faculty of Nursing, Ain Shams University, Cairo, Egypt

**Keywords:** Bullying, Prevention guidelines, Nurse interns, Assertiveness

## Abstract

**Background:**

Bullying is a serious problem that has short and long term negative consequences for nurse interns. Bullying prevention guidelines have a great impact on reducing the incidence of bullying among nurse interns.

**Aim:**

The present study aimed to evaluate the effect of developing bullying prevention guidelines on nurse interns’ assertiveness levels.

**Methods:**

A methodological study design was used to conduct the study at Ain Shams University Hospitals in Cairo Governorate, Egypt. The study subjects consisted of all nurse interns enrolled in the 2022–2023 internship year and their number 121 nurse interns. The data were collected using four tools: the Bullying Knowledge Questionnaire, the Negative Act Questionnaire Revised (NAQ-R), the Individual Bullying Behaviors in Clinical Practice Survey, and the Assertiveness Inventory.

**Results:**

The scores for total bullying knowledge and assertiveness after the implementation of the prevention guidelines were significantly increased (91.4% and 86.3%, respectively) among those who were exposed to bullying than among those with pretest scores of (34.7% and 11.8% respectively) (*P* < 0.001).

**Conclusion:**

Bullying prevention guidelines significantly reduced nurse interns bullying and improved their assertiveness. The study recommended the dissemination and generalization of the developed bullying prevention guidelines in different training settings.

**Supplementary Information:**

The online version contains supplementary material available at 10.1186/s12912-024-02128-z.

## Background

A successful internship period will smooth the transition and may yield a fruitful outcome in terms of enhancing graduates’ competency [1]. Academic internships are a bridge to link theory and practice by taking part in supervised and scheduled work. The challenges faced by nurse interns during internships affect their overall health and disturb their learning process. Nurse interns encounter complex issues, obstacles, and complications that demand a high level of skills that affect their practice [2, 3].

According to operational definitions, an internship is a student-focused learning experience related to a certain academic study field. In other words, internships provide students with practical experience in real-life situations that involve actual patients and incorporate some attitudes, values, and beliefs related to professional practice. Bullying is an aggressive act that manifests in various patterns of relationships with one’s surroundings [4]. Bullying is aggressive treatment by someone to hurt another person, is performed repeatedly, and aims to show the strength of the perpetrator to the victim [5]. Various types of bullying can range from verbal, social to physical bullying, such as kicking, hitting and other physical incidents [6].

The negative consequences of bullying for both victims and bullies have been well documented. In general, bullying victims are at risk of emotional and social consequences that are detrimental to nurse interns who are victims of bullying and who express feelings that lead to depressive symptoms; moreover, with low assertiveness, nurse interns feel anxious due to coping with their peers [7]. Most recent studies have shown a relationship between bullying behavior and assertiveness, possibly because bullying leads to a lack of assertiveness and social competence for victims, and bullies may attempt to display more extreme difficulty engaging in cooperative decision-making with their friends, which negatively affects their performance. In addition, overt dominance in their relationships with their victims’ university must improve nurse interns’ assertiveness to overcome bullying behavior experienced by nurse interns in their clinical practice areas, and bullying prevention guidelines are needed to eliminate bullying behavior in an assertive way [8].

On the other hand, assertiveness can be defined as a healthy way of communication and the ability to speak up about oneself in a way that is honest and respectful [9]. Assertive people care about other people’s feelings and therefore phrase their requests or compliance in a polite but firm manner [10]. Assertiveness is a fundamental learned interpersonal communication skill that helps individuals meet social demands [11]. An assertive person is crucial for both personal and organizational aspects. Assertive communication is advantageous in social, domestic, and professional contexts [12]. Assertiveness is particularly helpful for addressing uncomfortable or delicate situations. Using an assertive communication style helps nurse interns earn the respect of people and are able to communicate more effectively for career success and happiness [13].

### Significance of the study

Nurse interns are particularly vulnerable to real life situations because they are often younger, have less clinical and life experience, have fewer acquired coping skills, and have minimal power in unfamiliar environments [14]. Moreover, nurse interns are exposed to different sources of bullying, such as patients, relatives, peers, nurses, and other professional groups [15]. Bullying can be expressed in many ways, ranging from verbal aggression and excessive criticism or monitoring of work to social isolation. It has a negative effect on nurse interns’ performance and may lead to increased errors and decreased quality of nursing care [15].

Research on bullying behaviour for nurse interns in the clinical setting has shown that bullying has a tremendous influence on nurse interns assertiveness because they are often younger, they have less clinical and life experience, they have fewer coping skills, and they have minimal power in the work environment hierarchy. This has great negative consequences for behaviors that threaten performance on health care teams [16]. According to the World Health Organization (WHO), Physical fighting and bullying are also common among young people. A study of 40 developing countries showed that an average of 42% of males and 37% of females were exposed to bullying. [17].

There is great variation in the prevalence rates of bullying among different countries, but the rate among the Arab world is the highest [18]. The prevalence of bullying in Egypt was 77.8% among adolescents. Among these, the percentage of bully-victims was 57.8%, which can be explained by the likelihood of victims turning into bullies to express their anger [19]. Additionally, another Egyptian study reported that the prevalence of physical violence was the highest, at 69% for victimization and 82.8% for witnessing violence. There is a need to develop a deeper understanding of this problem and possible interventions for its treatment and prevention. Therefore, this study aimed to implement and evaluate the effects of developing bullying prevention guidelines for nurse interns and its effect on their assertiveness.

### Study aim

This study aimed to evaluate the effect of developing bullying prevention guidelines on nurse interns’ assertiveness levels. The studies hypothesized that the implementation of bullying prevention guidelines will increase nurse intern’s knowledge and attitudes regarding bullying behavior and significantly improve their assertiveness level.

## Methods

### Study design

A methodological design was utilized to achieve the aim of this study. The study subjects consisted of all nurse interns enrolled in the 2022–2023 internship year during the data collection period. A total number of 121 nurse interns were involved. The study was conducted at Ain Shams University Hospitals, Egypt. The hospital has 1,568 beds, and Ain Shams Main University Hospital is the city’s major teaching hospital, the Cairo governorate. The inclusion criteria for the research participants were as follows: were older than 18 years old, were nurse interns from the current graduation year 2022–2023, and voluntarily participated in the study. The exclusion criteria included nurse interns from old graduation years and who had spent their internship year.

### Instruments

#### Tool 1: personal characteristics of the nurse interns

Were compiled by the researchers and included questions about age, gender, marital status, place of residence, pre university education, training area and previously attended bullying prevention work shops.

#### Tool 2: the nurse interns’ knowledge bullying questionnaire

Was developed by researchers based on a literature review [20–22] to assess nurse interns’ knowledge regarding bullying before and after guideline implementation. The questionnaire was designed in the Arabic language to avoid misunderstanding. It is composed of 30 items: divided into two sections as follows: **Section (I)**, which includes 18 multiple choice questions and is categorized into five subscales: the concept of bullying and related definitions (4 items), types of bullying behavior (6 items), consequences of bullying behavior (4 items), and strategies and preventive measures to reduce the risk of bullying (4 items). **Section [II]**: This section includes 12 true/false questions covering different aspects of bullying. For each question, a correct response received a score of 1, and an incorrect response received a score of zero. These scores were converted into percent scores. Knowledge was considered satisfactory if the percent score was 60% or more and unsatisfactory if it was less than 60% [23]. The internal consistency reliability for the questionnaire assessing nurse interns’ knowledge of bullying was 0.914.

#### Tool 3: the negative acts questionnaire revised (NAQR)

This tool was adopted from Einarsen [24]. Twenty-two items were classified into three subscales as follows: work related bullying (7 items), person related bullying (12 items), and physical intimidation bullying (3 items). The scores ranged from “never” to “always” based on a 4-point Likert scale. A score of (< 60%) indicated a low level of nurse interns’’ experience of bullying, (≥ 60- ˂75%) indicated a moderate level of nurse interns’ experience of bullying and (≥ 75%) indicated a high level of bullying for nurse interns’ [25]. The internal consistency reliability for the revised negative act questionnaire was 0.913.

#### Tool 4: individual bullying behaviors in clinical practice survey

This survey was adopted from [26]. It consisted of 24 items. The scores ranged from “never” to “always” based on a 3-point Likert scale and the following variables were evaluated: daily experience of workplace bullying ≥ 75%, occasional experience of workplace bullying 60-˂75% and < 60% indicating a low level of workplace bullying experienced by nurse interns’ [26].The internal consistency reliability for individual bullying behaviors in the clinical practice survey was 0.798.

#### Tool 5: assertiveness inventory

This inventory was developed by [27] and adopted from [28]. It consists of 56 items classified into five subscales: verbal and nonverbal style (8 items), active participation (7 items), work habits (8 items), control of anxiety and fear (20 items) and relating coworker (13 items).The scores ranged from “never” to “always” based on a 3-point Likert scale. The items were evaluated as follows: highly assertive if the total score was more than 75%, moderately assertive if the total score ranged between 60 and 75% and low assertive if the total score was less than 60% [28]. The reliability of the assertiveness inventory was tested previously, and the score was 0.88 [28].

### Data collection phases

Official permission from the faculty of nursing at Ain Shams University, Egypt, Cairo, was obtained to conduct the study. The pilot study was conducted with thirteen nurse interns to examine the clarity, practicability, and feasibility of the tool and to estimate the amount of time needed to complete the study tools, which ranged from 30 to 45 min. The data obtained from the pilot study were analyzed, and no modifications were made. Therefore, the participants in the pilot study were included in the main study sample.

The data were collected for six months from the beginning of October 2022 to the end of March 2023. On October 2023, a pretest was performed, after which the researchers, from November to the end of December 2022, prepared the bullying prevention guidelines based on the analysis of the pretest data and determined the nurse interns needs. The researchers prepared the bullying prevention guidelines and returned them to the jury group to assess their accuracy, clarity in language (face validity), and importance of the items included in the guidelines (content validity). The jury group consisted of nine experts (professors and assistant professors) in the field of nursing administration and mental health nursing departments affiliated to Ain Shams, Damnhour, and Menoufia Universities, Cairo, Egypt.

### Bullying prevention guidelines

This phase of guideline design is based on the assessment data collected. The bullying prevention guidelines aimed to enhance nurse interns’ knowledge regarding bullying and how to prevent exposure to bullying and, and how to deal with it. The guideline booklet included theoretical content in the Arabic language to avoid misunderstanding and covered the following content: an overview of the internship year, the rights and responsibilities of nurse interns; bullying and related concepts, different types of physical, verbal, cyber and, social bullying and how to prevent the occurrence of each type of bullying and laws, legislation to reduce the incidence of bullying.

This phase of guideline implementation started at the beginning of November 2022 and the end of December 2022. The researchers disseminated instruction guidelines to all nurse interns and distributed a copy of the Bullying Prevention Guidelines booklet and explained it equally to each nurse intern. The researchers determined the specific time needed to meet the participants who needed additional clarifications about the guidelines. Each meeting lasted between 20 and 30 min; the meetings were conducted at nurse interns in the clinical setting to allow each participant to implement the developed guidelines during the clinical training period.

The posttest started from the beginning of February to the end of March 2023. After all the meetings with nurse interns were completed to ensure their understanding of the instruction guidelines, the researchers collected data for assessing the effect of designing and implementing bullying prevention guidelines for nurse interns and its effect on their assertiveness.

### Ethical considerations

The study was approved by the Research Ethics Committee, Faculty of Nursing/Ain Shams University, Egypt, Cairo [Code number: 23.9.128], based on the standards of the committee, Faculty of Nursing/Ain Shams University. An official letter containing the title and the aim of the study was sent to the director of each hospital to obtain approval for collecting data in the abovementioned settings. Nurse interns were assured that all data would be used for research purposes only, and each one was informed of the right to refuse participation in the study or withdraw at any time before completing the study tools with no consequences. Informed written consent was obtained from participants who agreed to participate in the study. Anonymity was considered and respected. Data confidentiality was assured during the implementation of the study.

### Statistical analysis

Data entry and statistical analysis were performed using the SPSS 20.0 statistical software package. The data are presented as frequencies and percentages for qualitative variables. The chi-square test was used to compare the qualitative variables which were also used to examine the relationships between two qualitative variables. Statistical significance was set at a p-value < 0.05. High statistical significance was indicated by a p-value < 0.001.

## Results

### Personal characteristics of the study subjects

Table 1 shows that 83.5% of the studied nurse interns were less than 24 years old, 52.1% were females, 76% were single, 34.7% were trained at Ain Shams University Hospital, 81% had secondary education before entering faculty, and 79.3% of the studied nurse interns had not previously attended training courses about bullying prevention before the current study.

**Table 1:** Personal characteristics of the study subjects

Nurse interns (*n* = 121)	No.	%
**Age**		
< 24 years	101	83.5
≥ 24 years	20	16.5
Mean ± SD	23.80 ± 0.94	
**Gender**		
Male	58	47.9
Female	63	52.1
**Marital status**		
Single	92	76
Married	29	24
**Training hospital**		
Ain Shams University	42	34.7
Aldemerdash	31	25.6
Pediatric University	24	19.8
Academic Heart Institute	24	19.8
**Previous education before faculty entrance**		
Secondary school	98	81
Technical nursing institute	23	19
**Previous attending of workshops about bullying prevention**		
Yes	25	20.7
No	96	79.3

### Nurse interns’ knowledge related to bullying before-after implementing guidelines

Table 2 shows the changes in the scores of nurse interns’ bullying knowledge, indicating a highly statistically significant improvement in nurse interns’ knowledge after implementing the guidelines compared with before developing the guidelines.

**Table 2:** Nurse interns’ knowledge related to bullying before- after implementing guidelines (*n* = 121)

Dimensions	Time					
	Before developing guidelines		After implementing guidelines			
	No.	%	No.	%	χ2	*P*
Concepts of bullying	36	29.8	114	94.2	86.43	< 0.001
Types of bullying	38	31.4	101	83.5	78.8	< 0.001
Consequences of bullying	44	36.4	100	88.5	84.74	< 0.001
Strategies and preventive measures	47	38.8	108	89.3	77.83	< 0.001
General knowledge	39	32.2	103	91.4	75.65	< 0.001
Total bullying knowledge	42	34.7%	103	91.4%	79.25	< 0.001

*p* < 0.001 was considered highly statistically significant

### Nurse interns’ negative acts before- after implementing guidelines

Table 3 shows the changes in the score of nurse interns’ negative acts, which was the highest percentage before the development of guidelines for the “work related bullying dimension”. This percentage declined to 12.4% after implementing the guidelines indicating a statistically significant improvement in reducing nurse interns’ negative acts (*p* < 0.001).

**Table 3:** Nurse interns’ negative acts before- after implementing the guidelines (*n* = 121)

Dimensions	Time					
	Before developing guidelines		After implementing guidelines			
	No.	%	No.	%	χ2	*P*
Work- Related Bullying	104	85.9	15	12.4	37.80	< 0.001
Person - Related Bullying	98	80.9	11	9	53.28	< 0.001
Physically Intimidation Bullying	89	73.5	17	14	72.67	< 0.001

*p* < 0.001 was considered highly statistically significant

### Nurse interns’ negative acts level before- after implementing guidelines

**Fig. 1:**
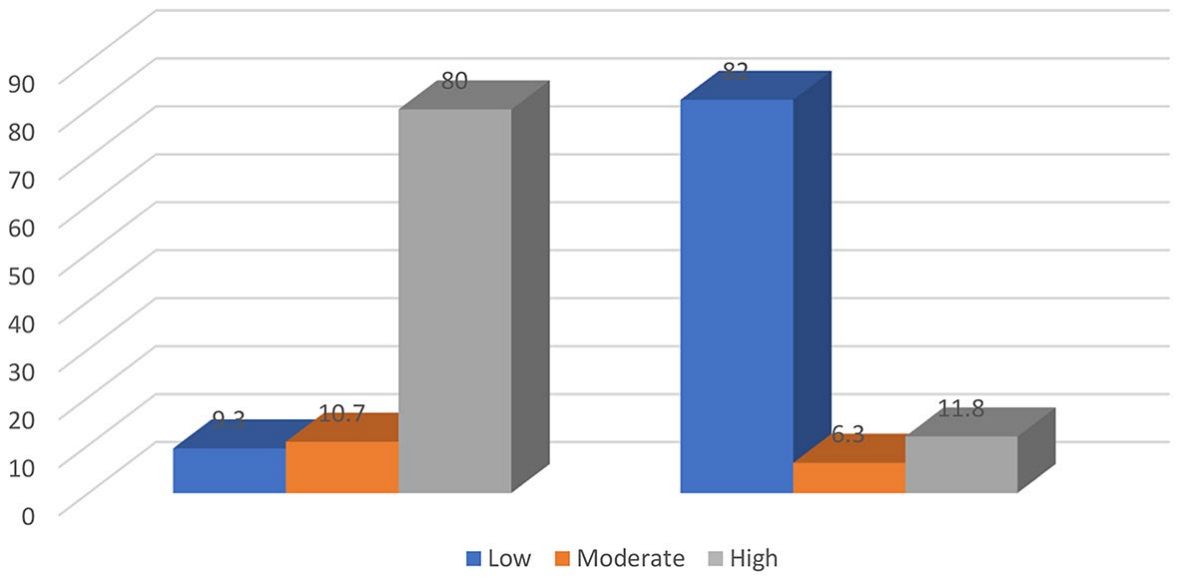
Nurse interns’ negative act levels before- after implementing the guidelines (*n* = 121)

Figure 1 shows that there was a highly significant reduction in negative acts after implementing guidelines among nurse interns’ compared with before developing guidelines (*p* < 0.001).

### Nurse interns’ individual bullying in clinical practice survey (sources of bullying) before- after the implementation of guidelines

Table 4 shows that the most common source of bullying before developing guidelines was staff nurses. Moreover, this percentage declined to 18.1% after the implementation of the guidelines.

**Table 4:** Nurse interns’ individual bullying in clinical practice survey (sources of bullying) before- after implementing the guidelines (*n* = 121)

Sources of bullying	Time					
	Before developing guidelines		After implementing guidelines			
	No.	%	No.	%	χ2	*P*
Staff Nurses	84	69.4	22	18.1	32.42	< 0.001
Clinical Instructor	18	14.9	5	4.1	90.36	< 0.05
Classmates	62	51.2	10	8.2	65.23	< 0.001
Physicians	11	9	4	3.3	88.32	< 0.05
pt./family members	80	66.1	15	12.3	46.14	< 0.001

*p* < 0.001 was considered highly statistically significant

*P* < 0.05 was indicate statistical significance

### Nurse interns’ assertiveness level before- after implementing guidelines

**Fig. 2:**
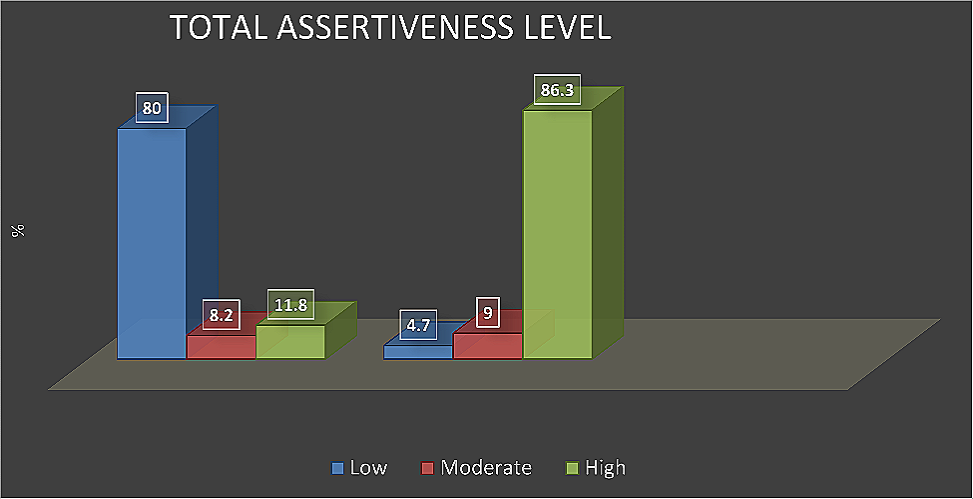
Nurse interns’ assertiveness level before- after implementing the guidelines (*n* = 121)

Figure 2 Illustrates changes in score of nurse interns’ assertiveness, indicating a highly statistically significant improvement in their assertiveness level after implementing guidelines compared with before developing guidelines (*p* < 0.001).

### Relationship between nurse interns’ bullying knowledge and negative acts

Table 5 Elaborates that there was a statistically significant relationship between the studied nurse interns’ bullying knowledge and their negative act after the implementation of the guidelines (P = < 0.001).

**Table 5:** Relationships between nurse interns’ bullying knowledge and negative acts (*n* = 121)

	Total Knowledge							
	Before developing guidelines				After implementing guidelines			
	Unsatisfactory (*N* = 79)		Satisfactory (*N* = 42)		Unsatisfactory (*N* = 10)		Satisfactory (*N* = 111)	
Negative Act	N	%	N	%	N	%	N	%
Low	7	8.9	32	76.2	1	10	93	83.8
Moderate	8	10.1	3	7.2	2	20	15	13.5
High	64	81	7	16.6	7	70	3	2.7
Test of significance	(**Χ2**) = 1.021 (P)= -0.207				(**Χ2**) = 46.301 (P) = < 0.001			

*p* < 0.001 was considered highly statistically significant

### Relation between nurse interns’ bullying knowledge and their assertiveness

Table 6 Elaborates that there was a statistically significant relationship between the studied nurse interns’ bullying knowledge and their assertiveness after the implementation of the guidelines (< 0.05).

**Table 6:** Relationships between nurse interns’ bullying knowledge and their assertiveness (*n* = 121)

	Total Knowledge							
	Before developing guidelines				After implementing guidelines			
	Unsatisfactory (*N* = 79)		Satisfactory (*N* = 42)		Unsatisfactory (*N* = 10)		Satisfactory (*N* = 111)	
Assertiveness level								
Low	63	79.7	13	31	2	20	4	36
Moderate	7	8.9	2	4.8	1	10	7	6.3
High	9	11.4	27	64.2	7	70	100	90.1
**Test of significance**	**(Χ2) = 1.230 (P)= -0.721**				**(Χ2) = 79.591 (P) = < 0.05**			

*P* < 0.05 was indicate statistical significance

## Discussion

Bullying behavior may lead to a lack of assertiveness and social competence for victims and bullies may attempt to display more extreme difficulty engaging in cooperative decision-making with their friends, negatively affecting their performance and assertiveness for both, as well as overt dominance in their relationships with their victims [29]. Furthermore, improving nurse interns’ assertiveness is essential for overcoming bullying behavior in clinical practice. In this context, the current study aimed to develop bullying prevention guidelines for nurse interns and investigate their effects on their assertiveness.

The findings illustrated that there were changes in nurse interns’ bullying knowledge scores, indicating a statistically significant improvement in their knowledge after implementing the guidelines compared with before developing the guidelines. This bullying is a major concern because it affects the physical and psychological health of nurse interns, and more studies about bullying prevention guidelines need to be included in the curriculum to help students become aware of this important topic. They can contribute to the improvement of nursing clinical learning and decrease the gap between theory and practice. This result agreed with that of Albayrak, Yıldız, and Ero [30], who reported that the participants showed a statistically significant increase in their knowledge regarding bullying after the implementation of the program. These results are consistent with those of Gaffney, Ttofi, and Farrington [31], who reported that the majority of the participants in their study had satisfactory knowledge regarding bullying after the program was implemented.

### Regarding negative acts among nurse interns

The present study revealed that there was a change in the score of negative acts among nurse interns, which was the highest percentage before receiving guidelines for the “work related bullying dimension”. This percentage significantly decreased after the implementation of the guidelines, indicating a statistically significant improvement in the reduction in negative acts among nurse interns after the implementation of the guidelines compared with before the guidelines were developed. This might be because nurse interns are often burdened with additional tasks and responsibilities, making them particularly vulnerable to becoming victims of workplace bullying, experiencing a workplace culture of silence, working extended hours, lacking clear anti-bullying policies, and lacking direct judgment regarding any bullying reports.

As mentioned by Ali, Keshk & Helal [32], who reported that work related bullying had the highest score on the negative act dimension before program implementation and a statistically significant improvement in reducing negative acts after program implementation compared with the pretest. These findings are generally congruent with the findings of Berry et al., [33], who reported the highest mean score for work-related bullying (M = 2.08, SD = 0.78), followed by person-related bullying (M = 1.99, SD = 0.73), while the lowest mean score was reported for physically-intimidating bullying. In contrast, Al- Garandeau, Laninga-Wijnen & Salmivalli [35], reported a statistically significant improvement in reducing negative acts after implementation of the program compared with before the program.

### Individual bullying behaviours in clinical practice survey

Concerning nurse interns sources of bullying throughout the study phases, the findings of the current study clarified that the most common source of bullying before developing guidelines was staff nurses. This percentage declined to 18.1% after the implementation of the guidelines. From the researchers’ perspective, staff nurses are usually in positions of power and authority and staff nurses most frequently contact nurse interns in clinical practice. In addition, nurses in hospitals commonly face high workloads, job demands, and long term high pressure environments, leading to heightened mental stress. Furthermore, the lack of experience of nurse interns who are afraid of responsibility and who have insufficient communication contributes to misunderstandings and bullying.

This result is consistent with that of Hopkins et al., [34], who reported that staff nurses were the first source of bullying in critical settings and that younger students were more likely to be bullied than older students. Similarly, Mostafa, Shazly & Hassan [37], reported that more than half of nurse interns consider staff nurses to be the most common source of bullying. Also, Additionally in contrast of a study conducted in Australia by Ullah et al. [3], who reported that more than half of dental interns considered teaching faculty including both junior and senior faculty members, to be the most common perpetrators of bullying.

### Total assertiveness level among nurse interns

The current study revealed changes in the scores of nurse interns’ assertiveness, indicating a statistically significant improvement in their assertiveness level after implementing guidelines compared with before developing guidelines.

This finding after implementing guidelines is supported by El-said, Shazly& Mostafa [35], who showed that the majority of the head nurses working at Ain-Shams University hospitals had statistically significant improvements in their assertiveness level at the post training program. Additionally, Nemati et al. [36], reported that the studied sample had a low assertiveness level before the training program while after the training program a statistically significant improvement in nurse interns’ assertiveness level was revealed. Moreover, in agreement with the findings of Mohamed [37–39], there were highly significant differences in assertiveness before and after training program implementation.

The current study revealed that there was a statistically significant relationship between the studied nurse interns’ bullying knowledge and their negative act guidelines and their assertiveness after the implementation of the guidelines. This relation indicates the comprehensiveness and effectiveness of the bullying guidelines on nurse interns’ assertiveness. This was observed in all areas, indicating the persistent effect of the guidelines. This improvement is certainly due to the direct effect of guidelines as indicated and the importance of providing updated knowledge about bullying prevention among nurse interns. The present results are in agreement with a study by [12], who reported that nurse interns bullying levels decreased, while their knowledge and assertiveness improved after the training program was implemented.

## Conclusion

The findings of the current study revealed that implementing bullying prevention guidelines for nurse interns was effective in improving their level of assertiveness in clinical practice during the internship year. Our findings revealed that nurse interns had insufficient knowledge about bullying in the pre-intervention phase and that there was a highly statistically significant improvement in nurses interns knowledge, behavior and assertiveness after implementing guidelines. The study recommended dissemination and generalization of the new and innovative bullying prevention guidelines to the different stages of nursing education, training nurse interns about dealing effectively with bullying behavior.

### Implications for practice and future directions

The findings gained from the current study can be valuable for using guidelines or programs for all academic and nonacademic organizations with the support of government authorities and applicable legislation. Universal application of prevention programs can increase awareness of the issue of bullying, creating a safe and positive environment for nurse interns where they can happily learn, grow and reach their full potential. Furthermore, awareness of bullying behaviors among nurses in hospitals where nurse interns are trained should increase. Moreover, the findings of this study will be beneficial for nurses, nursing educators, and clinical setting managers to design effective training programs according to the identified needs of nurse interns to facilitate the transition to a realistic clinical environment.

### Limitations of the study

First, one of the limitations of this study was the use of self-reports. All participants gained anonymity and confidentiality, but they still cannot completely avoid reaction bias. In addition, the sample size of this study was small, and included only nurse interns who were at Ain Shams University Hospitals in Cairo Governorate, Egypt. This limited the generalizability of the research results.

### Electronic supplementary material

Below is the link to the electronic supplementary material.

Supplementary Material 1

Supplementary Material 2

## Data Availability

Due to confidentiality concerns, the data and materials used in the current study cannot be made publicly available. However, they are available from the corresponding author upon reasonable request.
