# Supplementary material for: Developing bullying prevention guidelines for nurse interns’ and its effects on their assertiveness
Source: BMC Nurs. 2024 Jul 16;23:483. doi: 10.1186/s12912-024-02128-z (PMC11251234; doi:10.1186/s12912-024-02128-z)
Supplement: Supplementary file 1 — Supplementary Material 1 [file 12912_2024_2128_MOESM1_ESM.pdf]

Supplementary table (2): Reliability scores for the data collection tools:

Data Collection Tools	No. of items	Cronbach's Alpha
**1. Bullying knowledge questionnaire**	30	0.914
**2.Negative Act Questionnaire revised**	22	0. 913
**3.Individual Bullying Behaviors in clinical practice survey**	24	0. 798
