# Supplementary material for: Developing bullying prevention guidelines for nurse interns’ and its effects on their assertiveness
Source: BMC Nurs. 2024 Jul 16;23:483. doi: 10.1186/s12912-024-02128-z (PMC11251234; doi:10.1186/s12912-024-02128-z)
Supplement: Supplementary file 2 — Supplementary Material 2 [file 12912_2024_2128_MOESM2_ESM.pdf]

Supplementary table (1): Internal consistency of each dimension of the data collection tools:

Tools of data collection	No . of items	Cronbach's alpha	Standardized Cronbach's alpha	Mean item value	Mean inter-item correlation	Mean item total correlation
1. **Bullying knowledge questionnaire**	30	0.914	0.919	4.380	0.458	0.618
• Concept of bullying	4	0.911	0.915	3.907	0.537	0.718
• Types of bullying	6	0.933	0.937	4.111	0.668	0.771
• Consequences of bullying	4	0.852	0.855	3.881	0.501	0.680
• Strategies and Preventive Measures	4	0.910	0.913	4.455	0.622	0.779
• General knowledge related bullying	12	0.867	0.869	3.703	0.518	0.631
**2. Negative act revised questionnaire**	22	0.913	0.915	2.641	0.527	0.611
• Work related bullying	7	0.908	0.910	2.538	0.677	0.743
• Person related bullying	12	0.923	0.925	2.846	0.778	0.788
• Physical intimidation bullying	3	0.886	0.876	3.081	0.612	0.640
**3. Individual bullying behavior in clinical practice survey**	24	0.798	0.799	3.466	0.565	0.763
